# Aging of Superficial Musculoaponeurotic System of the Face—Novel Biomarkers and Micro-CT Relevance of Facial Anti-Gravity Support

**DOI:** 10.3390/diagnostics14111126

**Published:** 2024-05-29

**Authors:** Marius Valeriu Hînganu, Ramona Paula Cucu, Victor-Vlad Costan, Ludmila Lozneanu, Camelia Tamaș, Anca Elena Calistru, Liliana Hristian, Delia Hînganu

**Affiliations:** 1 Department of Morpho-Functional Sciences I, Faculty of Medicine, “Grigore T. Popa” University of Medicine and Pharmacy, 700115 Iasi, Romania; marius.hinganu@umfiasi.ro (M.V.H.); ludmila.lozneanu@umfiasi.ro (L.L.); hinganu.delia@umfiasi.ro (D.H.); 2 Department of Oral and Maxillo-Facial Surgery, “Grigore T. Popa” University of Medicine and Pharmacy, 700115 Iasi, Romania; victor.costan@umfiasi.ro; 3 Department of Plastic and Reconstructive Surgery, “Grigore T. Popa” University of Medicine and Pharmacy, 700115 Iasi, Romania; camelia.tamas@umfiasi.ro; 4 Department of Pedotechnics, “Ion Ionescu de la Brad” University of Life Sciences, 700490 Iasi, Romania; aecalistru@uaiasi.ro; 5 Department of Engineering and Design of Textile Products, “Gheorghe Asachi” Technical University of Iași, 700050 Iasi, Romania

**Keywords:** SMAS, anti-aging, facial ligaments, aging biomarkers

## Abstract

The soft superficial tissues of the face are against gravity through an intricate network of ligaments and ligamentous attachments. The aim of this investigation is to delineate the relationship between the muscular, fibrous, and vascular components of the superficial musculoaponeurotic system of the face (SMAS) at the level of its periosteal fixation areas from advanced radiological and novel biomarkers’ perspectives. These areas represent key points underlying skin aging and the longevity of restorative surgery results. Methods: This study was carried out on 37 surgical specimens, excised from patients admitted for surgery. On the excised specimens, we used special immunohistochemical techniques, such as markers for collagen type III, angiogenesis, vascular endothelium (I-CAM2) and muscle fibers (MYH2). We performed a micro-CT evaluation of these 37 specimens. Results: The results of this study showed different radiologic and IHC characteristics of the means of periosteal fixation of the SMAS. Evidence of morphohistological and radiological peculiarities of the retaining ligaments highlights new data for future functional studies of these structures. Our research must be continued with larger groups of subjects and through detailed methodological studies of vascular microperfusion and could represent an important new step in biotissue engineering and the customization of surgical techniques involving the sub-SMAS layers.

## 1. Introduction

The aspects of extrinsic aging of the face are based on the cellular senescence phenomena that appear with advancing age but also following exposure to favorable factors. It involves morphological and functional changes at the cellular level [1,2]. These changes begin to take place at the level of the SMAS anti-gravitational support mechanisms. The superficial musculoaponeurotic system (SMAS) is a complex structure that involves the superficial fascia, facial muscles, and adipose tissue. This structure works as a mechanism for distributing the force vectors of facial muscle contractions to the skin and fixes the superficial layers of the face anti-gravitationally. This occurs through the continuity of the superficial facial fascia with those of the surrounding regions, through its fixation to the periosteum, the tonus of the facial muscles as well as by the organization of the sub-SMAS adipose tissue [3,4].

Finding the ideal facial plastic was the goal of many plastic surgeons who had to deal with this type of intervention. The main requirements for this technique are the following:

- restoring a young, attractive appearance;
- an appearance that does not betray the surgical intervention;
- minimal complications;
- correction of all parts of the face;
- quick recovery;
- lasting results;
- and if possible, in accordance with current trends, the production of minimal and well-hidden scars.

Also, the main implications of these molecular phenomena are reflected in the incidents and accidents that occur when using various techniques for restoring facial appearances [5]. Most publications on this matter discuss, in particular, the level of dissection: the superficial plane, deep plan [6], extended SMAS, and subperiosteal [7,8].

Determining the zygomatic region as the pivot point for lifting the SMAS fold highlights the importance of this area. Good mobilization and superior lateral fixation of the SMAS affect the nasolabial folds and the sagging area of the cheek. It is considered that lifting and fixing the zygomatic fat by suturing is not necessary and is not feasible.

The most important principle of facelifting today remains subcutaneous dissection along with a generous SMAS dissection.

The classical anatomy of the SMAS includes the presence of a superficial subcutaneous fascia that invested the platysma muscle and fused with the external surface of the parotid fascia [9,10]. Subsequently, the consensus of surgical opinions seems to be that the SMAS represents the facial extension of the cervical fascia, enveloping the platysma in the neck and facial regions. In the upper part of the face, the SMAS passes over the zygomatic arch to continue with the superficial temporal fascia (temporoparietal) and the fascia of the fronto-occipital muscle [11,12].

For the functionality and homeostasis of these musculo-fascial structures, the means of periosteal fixation of the SMAS, mentioned above, intervene. They cancel the action of shear forces and gravity on the soft tissues of the face. In this sense, the muscles are fixed to the skeleton through the origin and insertion of the bone attachments. The adipose tissue and the skin receive some cutaneous extensions from the muscle fascia—the osteocutaneous ligaments, which pass directly from the dermis to the periosteum between the muscle origins. These are called retaining ligaments and include zygomatic, parotid, mandibulocutaneous, and zygomaticocutaneous ligaments (malar membrane) [13,14].

Most of the biomarkers studied so far reflect the specific situation of a biopsy area from multiple perspectives such as the qualitative examination of skin layers and the epigenetic and genomic aspects related to aging processes [15]. The evaluation of blood vessels in a tissue can be performed using basic histological techniques and staining with specific immunohistochemical markers (IHC) of the vascular endothelium. The expression of these IHC markers demarcates blood vessels because endothelial cells (EC) form their walls and perform a wide range of functions. They provide a selective barrier between blood flow and tissues and intervene in physiological processes such as thrombosis, angiogenesis, leukocyte traffic, and maintaining vascular tone [16]. Several studies have focused on the characterization of specific EC promoters [17,18] that are able to target endothelial cell gene expression in vivo and in vitro.

One of the first promoters shown to achieve uniform, high-level transgene expression in the vasculature of transgenic mice was that of the human intercellular adhesion molecule 2 gene (ICAM-2). This is a proteomic marker used until now to determine the aging phenomena that occur at the liver, acute myeloid leukemia, and lung levels [19]. In humans, this marker is a counter receptor for lymphocyte function-associated antigen-1 and can provide a costimulatory signal for T cell stimulation through the allogeneic major histocompatibility complex class II. Its expression is restricted to ECs and megakaryocytes [20] and is in vivo and in vitro.

Age-related neural impairments are closely associated with sarcopenia and muscle atrophy. Thus, the biomarker used in this study allows for the quantitative assessment of the presence of striated muscle fibers in the SMAS complex. In the case of skeletal muscle, the composition of fiber types influences physiological characteristics and susceptibility of the tissue to disease and aging. In their evaluation, anti-MYH2 antibodies (myosin heavy chain 2) are used in the immunodetection of the heavy chain of the myosin 2 protein [21,22]. In humans, this canonical protein comprises amino acid residues and its subcellular localization is in the cytoplasm. It expressed mainly in skeletal muscle and is directly involved in muscle contraction [23,24].

The third component of the SMAS, in addition to blood vessels, skeletal muscle fibers and nerve fibers, is connective tissue, represented at this level by type III collagen. Along with type I collagen, type III collagen forms the cellular matrix of the deep face of the dermis to which the SMAS adheres [25]. At the same time, this type of connective tissue forms septa and ligamentous adhesions that cross the SMAS and fix the dermis to the periosteum in certain regions and in different manners.

The skin’s extracellular matrix (ECM) plays a vital role in maintaining its physiology, and collagens are major components of the ECM. The quantitative and topographical determination of type III collagen fibers, correlated with their examination via micro-CT, can become an extremely useful biomarker in the determination of facial aging phenomena and the evaluation of medium- and long-term results of lifting interventions [26].

The simultaneous examination of IHC markers for blood vessels, skeletal muscle fibers, and type III collagen brings us a unitary, conclusive, and even reproducible approach to the modelling of the topographical and morphological organization of the SMAS retaining ligaments.

The aim of our study is to highlight the interrelationship between connective tissue and mimic muscles at the level of periosteal insertions and to relate these aspects to the features of the vascular apparatus of the SMAS. Using these anatomical data, we focus this study on the functional research of these adhesions that have a direct impact on the plastic and reconstructive surgery techniques for the face.

## 2. Materials and Methods

We proceeded with our study in three complementary stages.

### 2.1. Anatomical and Surgical Study

In the first stage, we used embalmed specimens in order to identify the manners of fixation of the SMAS to the zygomatic arches and the zygomatic and temporal as well as periorbital ligaments. This step allowed us to train for faster exploration and easy identification of them in the second stage of the study, namely the moment of surgical interventions.

The material used was represented by seven formalized cephalic extremities (14 pieces) and 37 operative pieces from the Oral-Maxillo-facial Surgery Clinic and the Plastic Surgery and Reconstructive Microsurgery Clinic of the Emergency Hospital St. Spiridon in Iasi.

On the 2% formalin-fixed specimens, a thorough dissection of the face was practiced, plane by plane and bilaterally. After a perpendicular incision in the dermis of the region of interest, we highlighted the fascial, adipose, and muscular planes and the fixation ligament formations. Both the anatomical and surgical dissection were focused on the insertion sites of the retention ligaments of the face.

Operative interventions allowed for anatomical parcellation studies according to the objective of the intervention, ensuring live visualization of some fascial and muscular structures and the possibility of dissociating the planes but also evaluations regarding their vascularization.

Before starting the histological study, we standardized the work technique to obtain objective and comparable results. In all cases, we followed a sampling technique with similar anatomical landmarks and incision methods:

- choosing the place of preservation of the samples to be analyzed and the collection method;
- the sampling of vascular fragments, in all cases, should be performed using the same technique;
- the tissue fragments should be fixed immediately, while avoiding their manual manipulation, as delays in the fixation process may affect the quantifications performed;
- the harvested fragments should be fixed in the same substance in order to obtain reproducible results and with a low rate of variation;
- a single laboratory technician, familiar with the requirements and processing techniques of the tissues, ensured the standardized processing of the parts;
- using the same computer program for the analysis of µCT images;
- the microscopic and radiological explorations were performed by the same persons in order to avoid subjective variations between several observers.

In the same preoperative stage, we performed the radiological identification of these fixation methods using the MRA technique.

The third stage consists of the histological study and IHC on the resection pieces from the level of these SMAS fixation methods.

### 2.2. Hystological and Imunohystological Experiment

Tissue samples containing fragments of the retaining ligaments of the facial superficial musculoaponeurotic system (SMAS) were stained using hematoxylin and eosin (HE) and Masson (van Gieson) trichrome (VG).

IHC (immunohistochemistry) protocols

Tissue blocks were taken from the parotid, maseteric, jugal, and zygomatic regions during surgical procedures for different pathologies that did not involve the quality of the SMAS tissues. The exclusion criteria consisted of facial malignancies of the skin and subcutaneous tissue and all types of dermatologic conditions or those arising after radiation therapy.

This study followed the principles outlined in the Declaration of Helsinki. Written informed consent was obtained from each participant who underwent MRA. The approval of the ethics committee of the Grigore T. Popa University of Medicine and Pharmacy, Iasi with number 195, and dated 3 June 2022 is attached to this manuscript.

In total, 37 facial fragments were excised for histological and immunohistochemical examination. The samples were stained with collagen III and ICAM-2 and MyoH2 antibodies. Each tissue section had a positive control (normal connective tissue elements, endothelial cells, and muscle fibers).

Formaldehyde-fixed human tissue was embedded in paraffin wax, then deparaffinized sections were incubated in phosphate buffered saline (PBS), and 4 μm sections were cut for IHC staining. Heat-induced epitope retrieval with citrate buffer, pH 6.0, was performed before peroxide blocking for 10 min and staining with type III collagen (clone FH-7A, Abcam, UK), 1/500 dilution, ICAM-2 (clone EPR 19114-113), 1/4000 dilution, Abcam, UK), MyH2 (clone A4.74, Abcam, UK), and 1/1000 dilution. Sections were developed using a mouse- and rabbit-specific HRP/DAB detection IHC kit with HRP conjugated secondary antibodies (biotinylated goat-anti polyvalent) for 10 min and streptavidin peroxidase, after which they were counterstained with hematoxylin to visualize collagen fiber morphology and endothelial, or muscular cells. Images of histological slides were taken at different magnifications with a camera attached to a light microscope.

The intensity of the immunohistochemical staining response for Collagen III (collagen type III), endothelial cells (ICAM-2), and muscle fibers (MyH2) was scored between zero and three. This represents a histological scoring system as an expression of the amount of collagen, epithelial cells, and muscle fibers as well as the intensity of staining. Our subjective qualitative assessment was as follows: (0) for negative results; (1) + small amounts/low intensity (weak); (2) ++ for moderate amounts/moderate intensity; and (3) +++ for large amounts/strong intensity) [27]. Each type of zone had a characteristic staining pattern for type III collagen, ICAM-2, and MyoH2.

*Micro-CT* (*µCT*) examination of the formalin-fixed paraffin embedded (FFPE) or just formali-fixed specimens was the fourth stage. µCT is a 3D radiological technique that uses X-rays to penetrate an object, slice by slice. It is also called microtomography or computerized micro-tomography, which is similar to a hospital CT or “CAT” scan but is on a small scale with a much higher resolution. Pixel sizes can be up to 100 nanometers, and the maximum possible diameter to scan is up to 200 mm. For the micro-CT study, we used pieces from the 14 formalized specimens and 23 of the surgical pieces. The 23 operating pieces were deparaffinized after the sections for the IHC slides were performed.

A series of 2D planar X-ray images were captured, and 2D cross-sectional data was reconstructed. These were further processed into 3D models and even printed as physical 3D objects for analysis. This allowed one to see inside the object and reveal its internal features and non-destructively gather volumetric data about the microstructure.

The X-ray source transmitted through the sample, and these were recorded by the X-ray detector as a 2D projection image. A fraction of a degree rotation on the rotary stage was then performed, and another X-ray projection image was taken. This step was repeated for 180 degrees (or sometimes 360 degrees, depending on the type of specimen). The series of images was subjected to “reconstruction” through algorithms and calculation formulas.

This study followed the principles outlined in the Declaration of Helsinki. This study protocol was reviewed and approved by Ethics Research Committee of Grigore T. Popa University of Medicine and Pharmacy Iasi with approval number 195 and dated 3 June 2022.

### 2.3. Quantitative and Statistical Evaluation of the Obtained Data

On the IHC slides, we applied a scoring for the expression intensity of the used marker. Thus, +1 means weakly positive values, +2 moderately positive, and +3 strongly positive.

Our study correlated these values with the metric measurements made on the micro-CT images using the software provided with the used equipment.

The verification of the results’ normality distribution obtained for each variable was carried out via graphical and statistical methods in the SPSS Statistics Version 20 program and using the Kolmogorov–Smirnov test and the Shapiro–Wilk test. This study includes the following:

Categorical independent variables (Collagen III, ICAM-2 and MyoH2);
Numerical independent variables, (TSMAS (µ), TAT (µ), BVD (µ), N (µ) and L (µ));
Dependent variable, anatomical region (pretracheal, periorbital, and preauricular).

The Shapiro–Wilk (S-W) test uses the null hypothesis that a sample comes from a normally distributed population. If the value of Sig. is less than a chosen alpha significance level or threshold (0.05), then the null hypothesis is rejected (i.e., it is unlikely to obtain such data assuming they are normally distributed) [28].

The Kolmogorov–Smirnov test is used to estimate the normality of a distribution where the mean and standard deviation can be calculated. It is used to verify the hypothesis that a data sample follows a certain distribution law as well as to compare the distribution laws of the populations from which two samples come [29].

The coefficient of asymmetry (Skewness) expresses the degree of displacement to the left or to the right of the distribution of a variable compared to a normal distribution.

The coefficient of curvature (Kurtosis) expresses the degree of grouping of the results around the central value.

## 3. Results

Blood vessels within the superficial soft tissues of the face form layers of parallel and interconnected vascular networks that supply fascia, subcutaneous tissue, and skin. Today, discuss the fascial plexus that is located deep in the deep muscle fascia and is supplied by musculocutaneous and septocutaneous vessels.

Another subcutaneous plexus lies within the SMAS. Branches from this area exit the superficial fascia and divide into subcutaneous fat in both superficial and deep layers. This plexus also consists of the musculocutaneous and septocutaneous arteries.

### 3.1. Hystological and IHC Studies

We collected the samples for study from topographical regions corresponding to the insertions of the zygomatic and temporal ligaments and the periorbital ligament adhesions, which are known to not contain blood vessels and nerve structures. These collection points were found to be pretragal (3 cm anterior to the tragus, corresponding to the lateral part of the zygomatic region), preauricular (3 cm anterior to the horizontal plane taken through the upper limit of the insertion of the auricle), and periorbital—1 cm laterally from the middle of the supraciliary arch and 1 cm laterally from the middle of the orbital rim and in the center of the zygomatic frontal arch.

**Figure 1:**
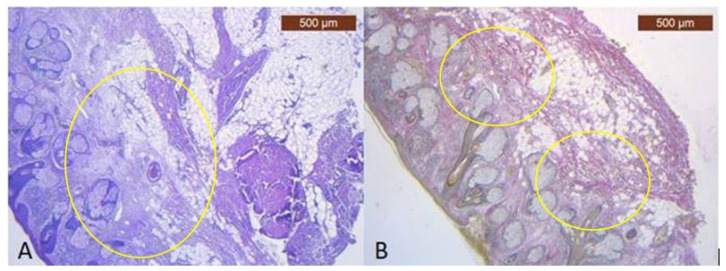
(**A**) Continuous musculo-adipose-fibrous network sending several extensions to the dermis (HE × 2.5); (**B**) Distinct fibro-adipose layer (VG × 2.5).

**Figure 2:**
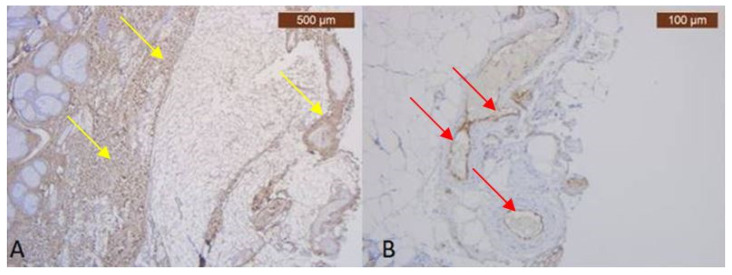
(**A**) Striking and strongly positive staining for Collagen III—yellow arrows (Anti-Collagen III × 2.5); (**B**) ICAM-2 mildly expressed on the blood vessels epithelium—red arrows (Anti-ICAM-2 × 10), which may be visible only in special IHC stain.

In the preauricular area, there are thin muscular fibers in its peripherals (Figure 1B) and negative staining in its center with the anti-MyH2 x20 antibody. This is why we have not included this slide but the expression of the ICAM-2 antibody in Figure 2 (Figure 2B). This means we have a lack of muscles, but blood vessels are visible using light microscopy.

**Figure 3:**
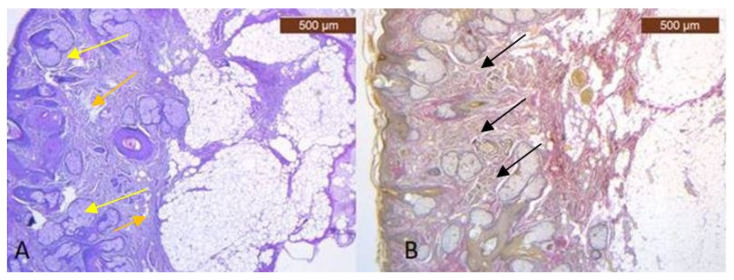
(**A**) Collagen (brown arrows) and adipose cells (yellow arrows) in similar proportions to the skin in deep dermis (HE × 5); (**B**) Superficial facial expression muscle fibers (black arrows) and adipose lobules extends to connect with the dermis of the skin (VG × 2.5).

**Figure 4:**
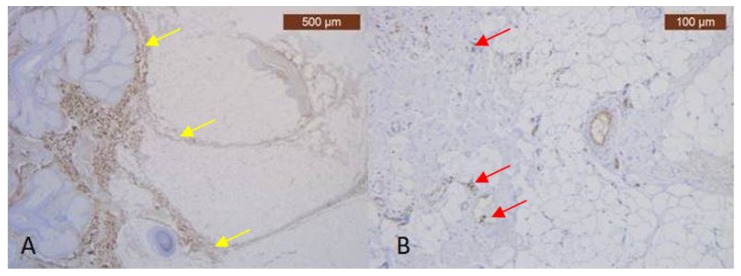
(**A**) In the deep area, the collagen appeared scanty relative to that present in surrounding superficial dermal connective tissue—yellow arrows (Anti-Collagen III × 2.5); (**B**) ICAM is absent except in capillary and thick blood walls, which were frequently positive. Blood vessels are found only at the periphery of the lamina and are very small in size—red arrows (Anti-ICAM-2 × 10).

In the pretragal area, the sub-SMAS adipose tissue is well represented (Figure 3A,B), while the muscle tissue is almost missing. Meanwhile, we also have the expression of the ICAM-2 antibody (Figure 4A,B).

**Figure 5:**
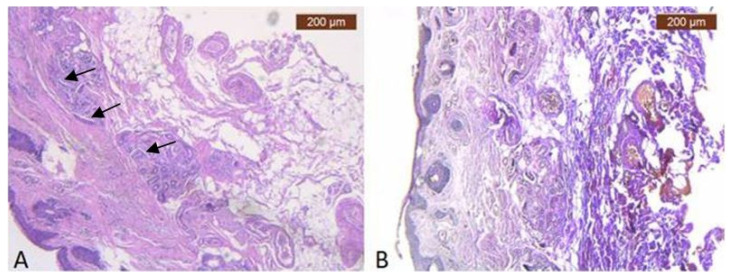
(**A**) Connective tissue layers. Nerve branches can be found in the fibrous septa in the deeper layer of the subcutaneous tissue (HE × 5)—black arrows; (**B**) Connective tissue layers. Fibrous septa in the deeper layer of the subcutaneous tissue (VG × 5).

**Figure 6:**
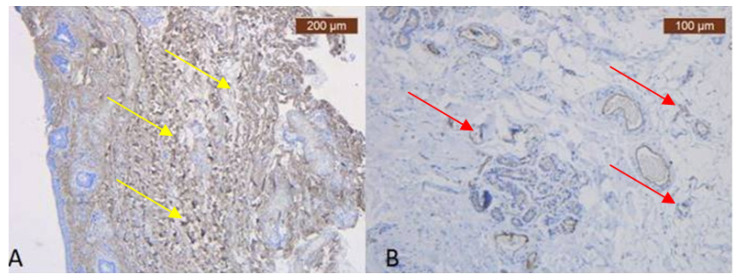
(**A**) Type III collagen more irregularly dispersed, in the dermis—yellow arrows (Anti-Collagen III × 5); (**B**) Details on negative area showing staining confined to blood vessels (Anti-ICAM-2 × 10). Even in this area there are thin blood vessels within SMAS, visible on special IHC marker—red arrow.

We have similar results in the infraorbital region. (Figure 5 and Figure 6).

**Figure 7:**
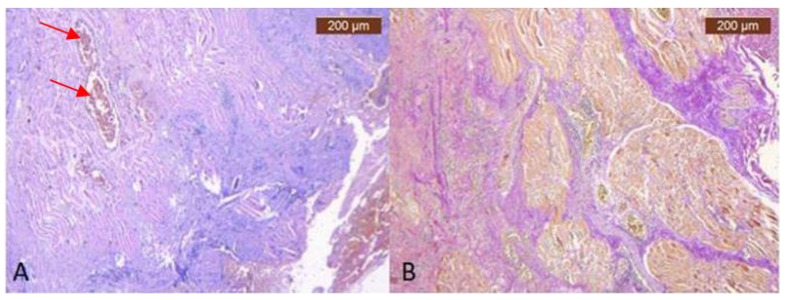
(**A**) Overview of temporo-frontal area, which consist of fibromuscular sheet of delicate collagen fibrils and muscle fibers containing the vasculature—red arrows mark IHC only visible in ensheathed blood vessels (HE × 5); (**B**) Deep vertical and horizontal collagen fibrous septa (red) and muscular fibers (yellow) (VG × 5).

**Figure 8:**
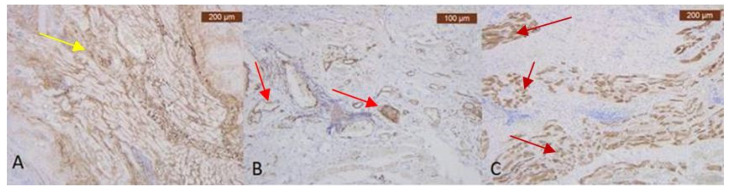
(**A**) Abundant perivascular and perimuscular type III collagen—yellow arrow (Anti-Collagen III × 5). Staining intensity: moderate; (**B**) ICAM-2 is expressed in blood vessels’ epithelium, with moderate and strong stain of endothelium—red arrows (Anti-ICAM-2 × 10). Staining intensity: weak and moderate, which highlights thin blood vessels within SMAS visible only using special IHC marker; (**C**) Immunohistochemically strong positive myosin reaction of muscular fibers in SMAS fibrous area—dark red arrows (Anti-MyH2 × 5). Staining intensity: moderate.

In the temporo-frontal area, there are muscular fibers, but the collagen ones are much thinner (Figure 7A,B). The IHC staining for the muscular cells is also positive, and the vascular marker signals the presence of a vascular network (Figure 8A–C).

### 3.2. Micro-CT Study

The results of our micro-CT study are consistent with those of IHC stainings. We were able to identify the dermal and subdermal vascular plexuses as well as the communication between them.

**Figure 9:**
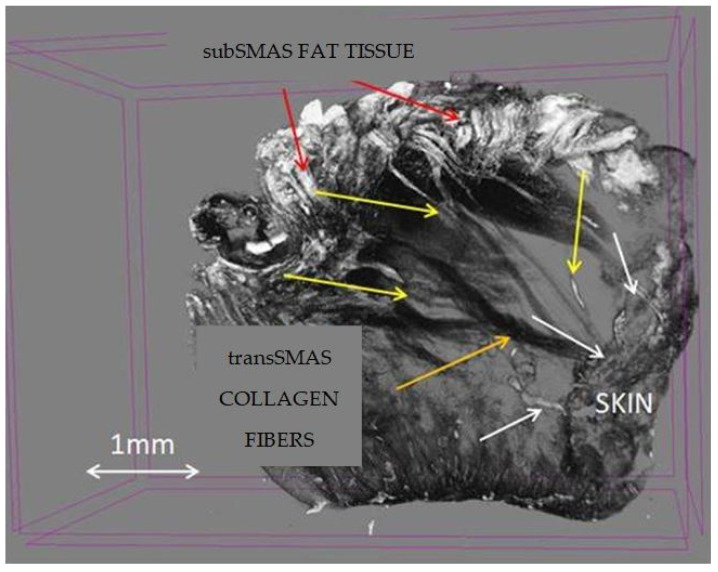
Micro-CT specimen after 7 days immersion in Lugol iodide solution. Pretragal area. 7 µm scanning; red arrows—subSMAS fat tissue; white arrows—skin; yellow arrows—blood vessels; orange arrow—collagen fibers.

**Figure 10:**
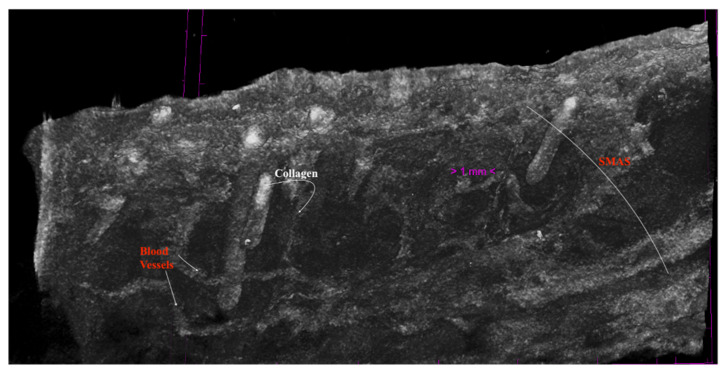
SMAS fibrous collagen septa crossing to the skin together with muscular fibers; transvers and tortuous blood vessels within SMAS. Preauricular area; specimen after 14 days in Lugol Iodide solution. 7 µm scanning.

In the preauricular area, we found blood vessels that are parallel with the skin within the SMAS thickness (Figure 9 and Figure 10).

**Figure 11:**
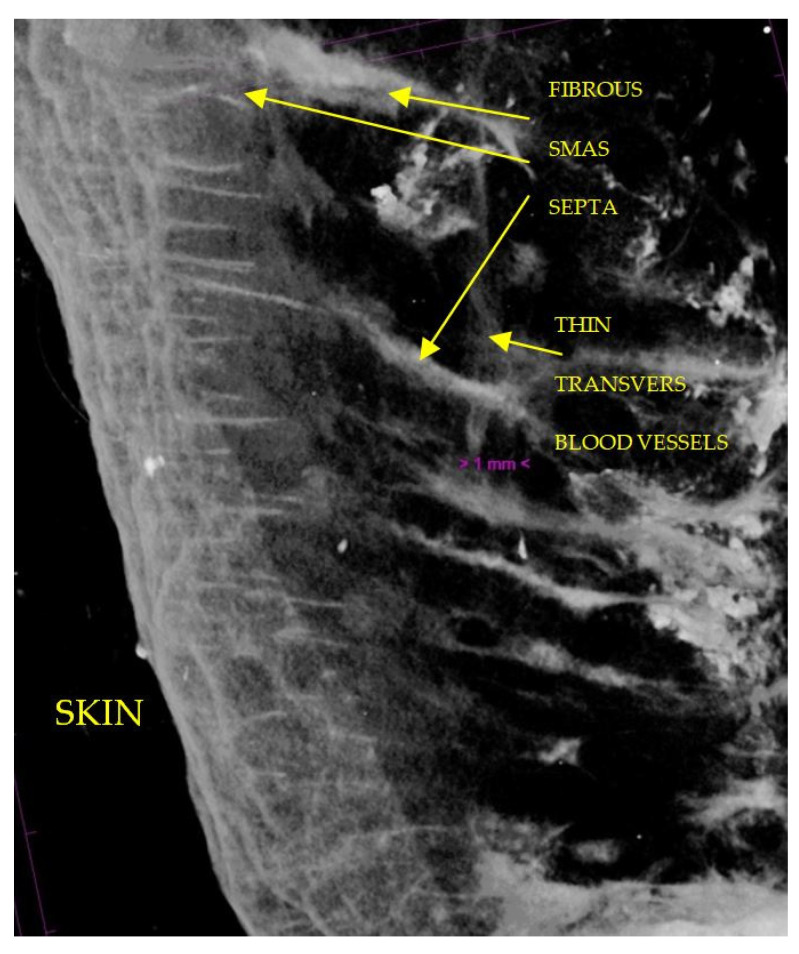
7 µm scanning of infraorbital region, rotated at 90° in order to avoid a large amount of adipose tissue and to see SMAS in detail; numerous collagen tracts of SMAS running into the skin; thin transvers blood vessels.

The infraorbital region shows the particular micro-CT features. Here, we have numerous collagen SMAS tracts that adhere to the skin and are crossed by thin blood vessels in a parallel manner (Figure 11).

**Figure 12:**
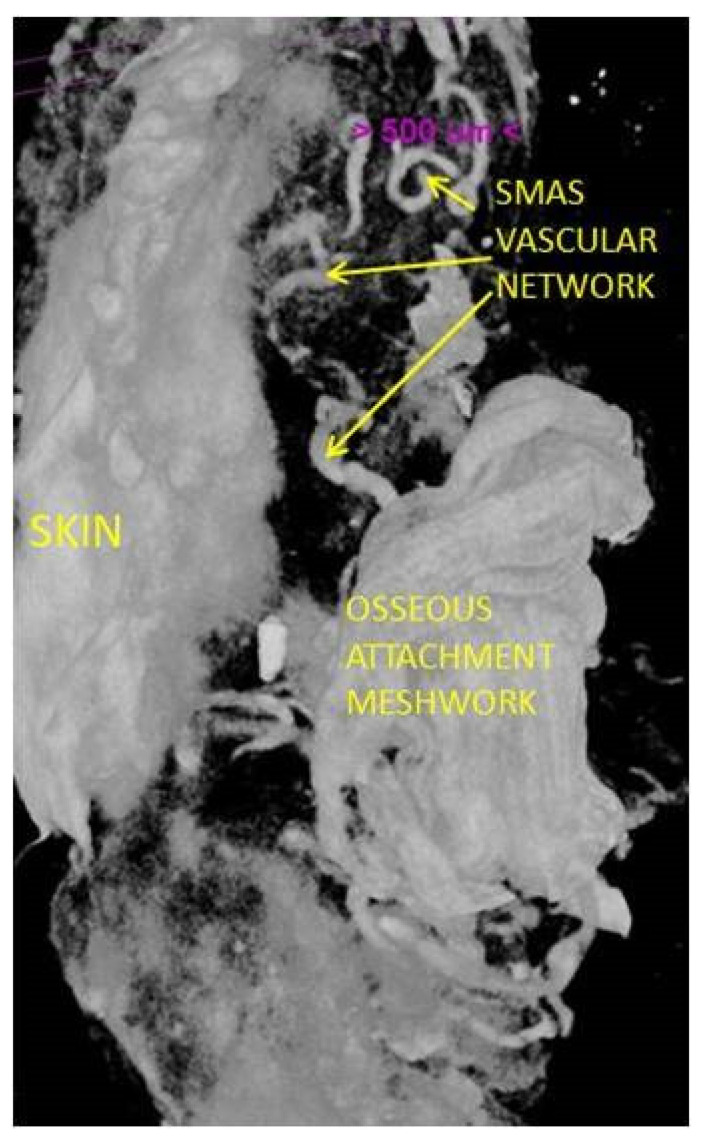
Well-represented vascular network and a strong collagen attaching meshwork; 14-day immersed specimen in Lugol iodide solution; 7 µm scanning.

In the examined supraciliar area, there is a rich vascular network within the SMAS. There are no visible muscular fibers in our section. The collagen fibers are so dense around blood vessels that we had to rotate the image 90° to be able to see them (Figure 12).

**Figure 13:**
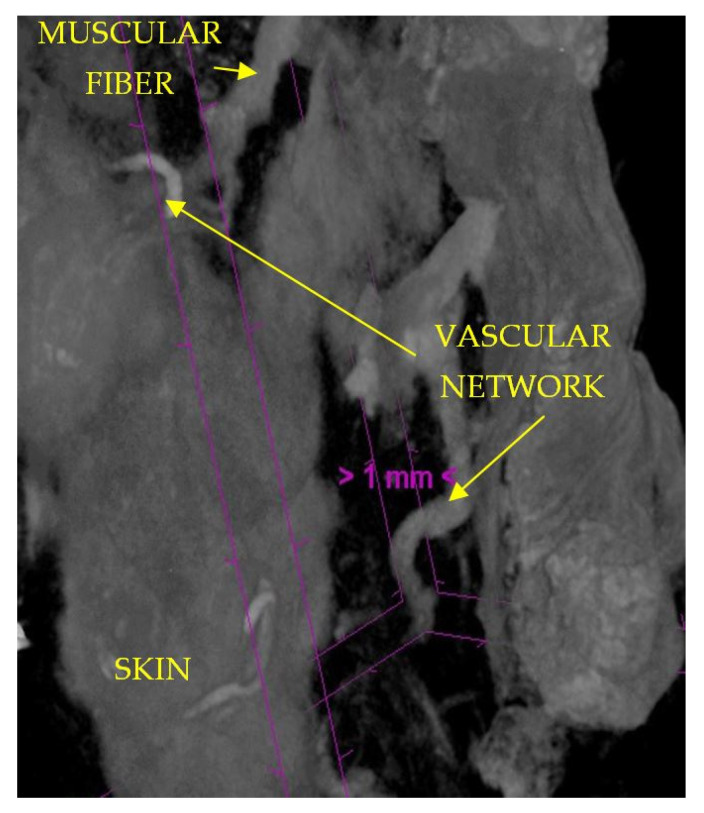
Moderate vascular network in temporo-frontal region; moderate muscular fibers in 7-day immersed specimen in Lugol iodide solution; 7 µm scanning.

The temporal frontal area shows individual SMAS features on micro-CT examination. There is a moderate vascular network within the SMAS together with distinctive collagen and muscular fibers (Figure 13).

### 3.3. Quantitative and Statistical Evaluation of the Obtained Data

By applying the Kolmogorov–Smirnov and Shapiro–Wilk statistical tests for the independent variable, Collagen III, the relevant results can be found in Table 1 regarding the descriptive statistics. We are interested in the coefficient of asymmetry (Skewness) and the coefficient of vaulting (Kurtosis), by which we can appreciate the shape of the distribution of the variables.

Table 2 contains the Kolmogorov–Smirnov (K-S) test statistic values, the number of degrees of freedom (df = 37), and the statistical confidence, Sig. 0.000. Because the statistical confidence Sig. 0.000 < 0.05, it follows that the test is statistically significant and the independent variables do not follow a normal distribution law.

Furthermore, by applying the Shapiro–Wilk (S-W) test, it can be seen that the statistical safety Sig. 0.000 < 0.05. It results that the test is statistically significant, and the variables in the study do not follow a normal distribution law.

Because, by applying the Shapiro–Wilk (S-W) test, the statistical certainty Sig. 0.000 < 0.05, normality tests indicate that non-parametric methods (such as the Kruskal–Wallis test) should be used to check whether the samples come from the same distribution. The Kruskal–Wallis test is thus used to compare two or more independent samples of equal or different sample sizes.

The mean rank (i.e., the “Mean Rank” column in Table 3) of the dependent variable “Anatomical region” for each group can be used to compare the effect of different groups. If these groups in the anatomical region have different scores for each independent variable, then it can be evaluated using the statistics table showing the results of the Kruskal–Wallis H test, presented in Table 4.

By applying the Kruskal–Wallis test, it is observed that the estimated value of the Chi-Square statistic for each independent variable is significant at a confidence level of 95% because the value of Asymp. Sig. < 0.05, with the exception of the variables ICAM-2 and MyoH2.

To decide whether the independent variables included in the study follow a normal distribution, we transformed the two coefficients into z-scores, as follows:

$$z_{Skewnes}=\frac{S}{{SE}_{skewness}}\;și\;z_{Kurtosis}=\sqrt{\frac{K}{{SE}_{Kurtosis}}}$$

where: S is the statistic of the Skewness coefficient, K is the statistic of the Kurtosis coefficient, SE_skewnes_ is the standard error of asymmetry, and SE_kurtosis_ is the standard error of the vault.

If one of the z scores thus obtained is

$$\left|z_{Skewness}\right|>1\;;\;\left|z_{Kurtosis}\right|>1\;\mathrm{or}\;\left|z_{Skewness}\right|>1,5;\;\left|z_{Kurtosis}\right|>1,5$$

(for small volume samples), then the distribution differs significantly from a normal distribution.

According to this algorithm, the values obtained for each independent variable are centralized in Table 5, where it can be observed that at least one of the z scores thus obtained is $\left|z_{Skewness}\right|>1$ or $\left|z_{Kurtosis}\right|>1$, which leads to the conclusion that the distribution of the variables differs significantly from a normal distribution.

**Figure 14:**
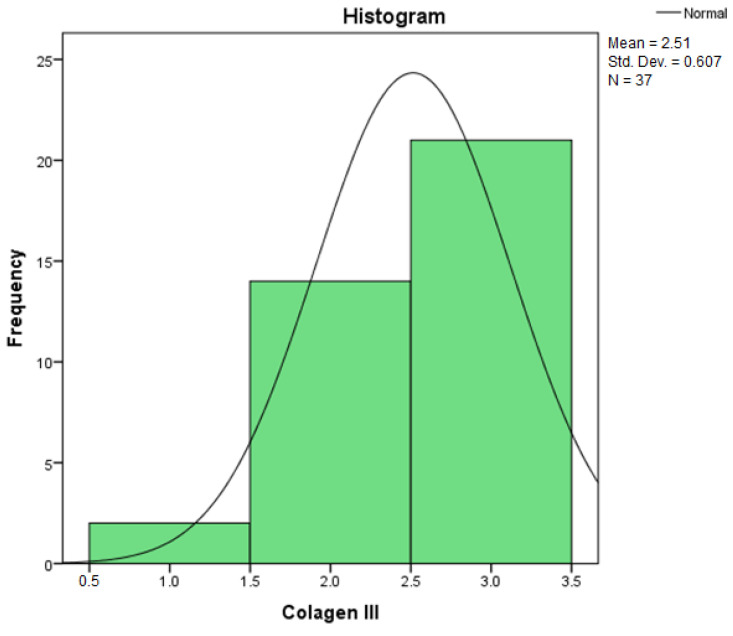
Histogram for the variable Collagen III.

**Figure 15:**
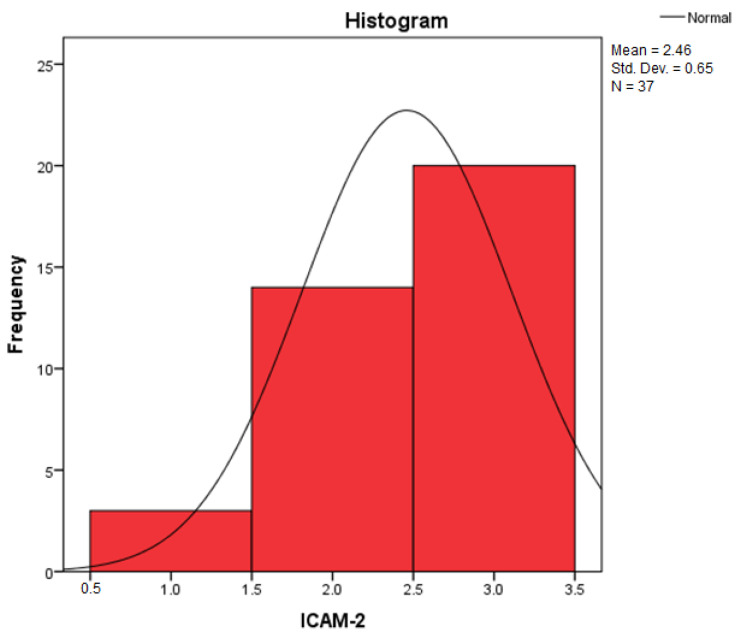
Histogram for the variable ICAM-2.

**Figure 16:**
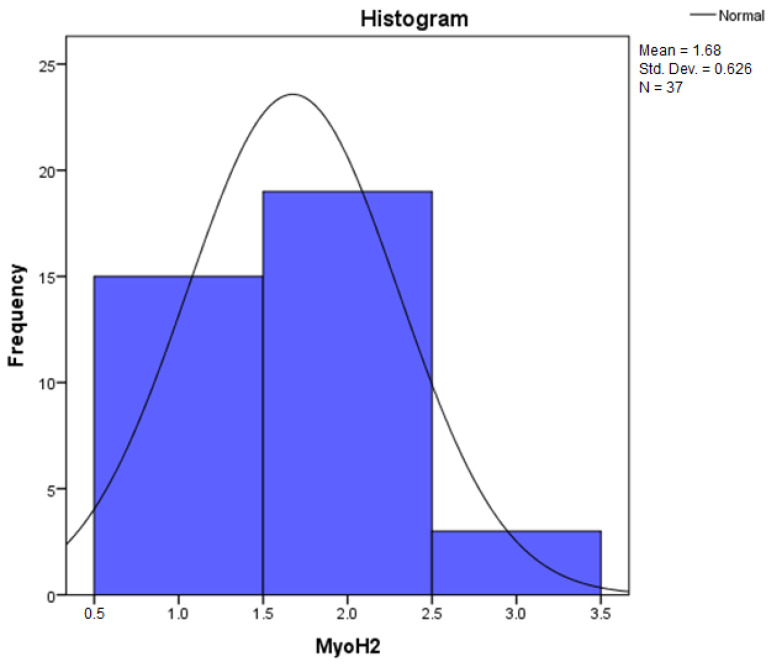
Histogram for the variable MyoH2.

**Figure 17:**
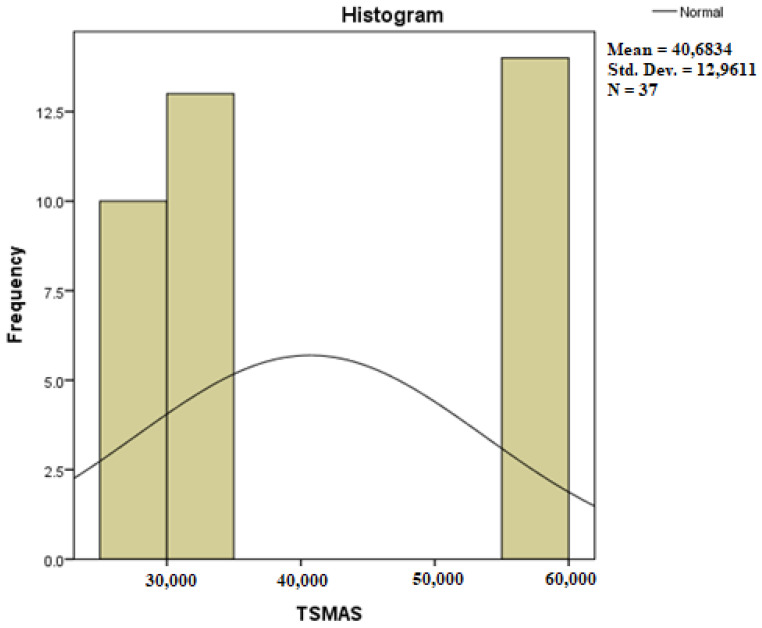
Histogram for the variable TSMAS.

**Figure 18:**
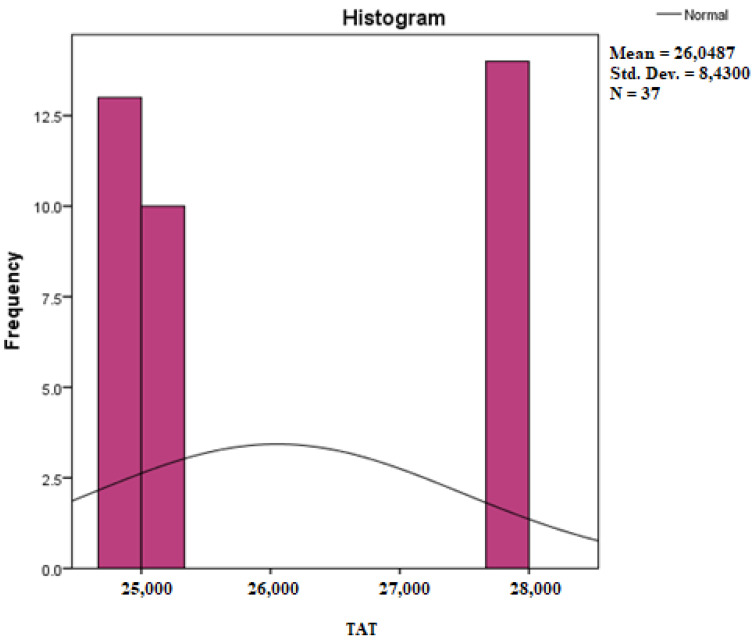
Histogram for the variable TAT.

**Figure 19:**
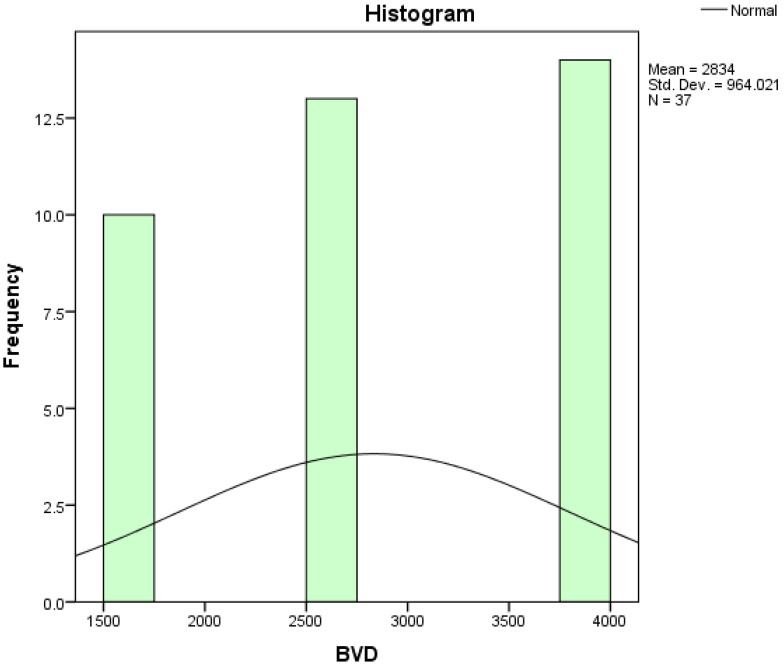
Histogram for the variable BVD.

**Figure 20:**
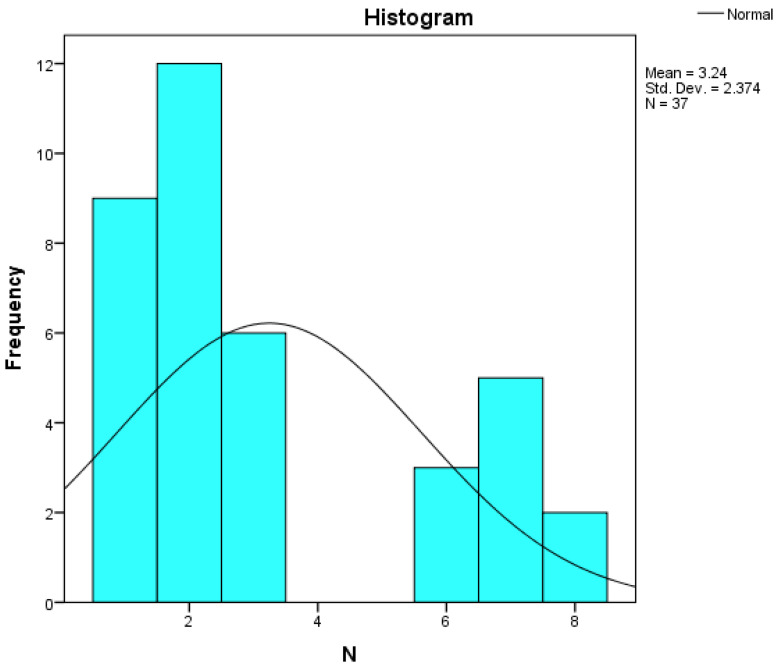
Histogram for the variable N.

**Figure 21:**
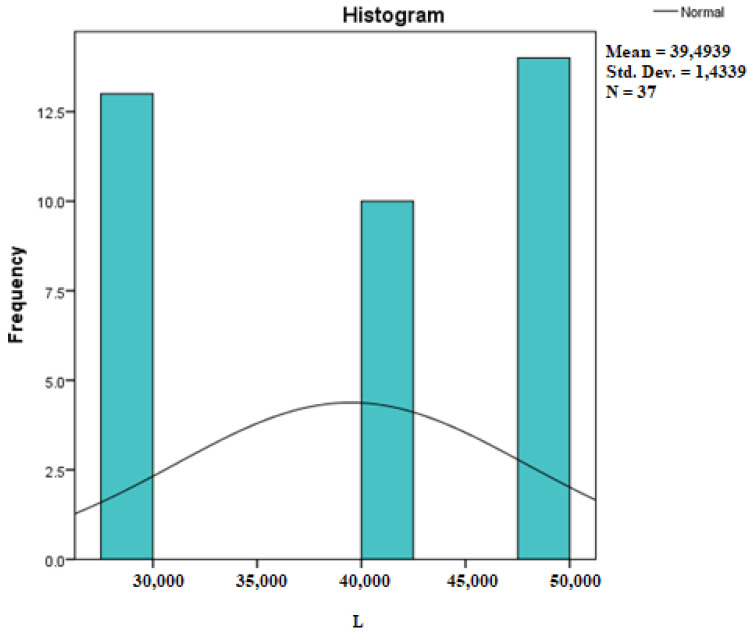
Histogram for the variable L.

Assessing the normality of the distribution of the results obtained for the variables included in this study was also undertaken using graphical methods. For the graphical method, the most common technique is displaying the data as a histogram, illustrated in Figure 14, Figure 15, Figure 16, Figure 17, Figure 18, Figure 19, Figure 20 and Figure 21, where it can be observed that the frequency distribution of the data for each variable does not follow a normal distribution law, being characterized by an asymmetry to the left or to the right.

## 4. Discussion

The initial description of the arterial blood supply of the SMAS and of the skin stipulates that it originates in two networks. This commenced prior to the inception of the SMAS as a concept and is still accepted nowadays [30,31,32,33,34]. The main network, formed by deep muscular perforators, anastomoses into a subaponeurotic vascular arch. Its ramifications are perpendicular to the skin plane and join a subdermal vascular arch. Another network corresponds to direct cutaneous arteries [35].

Pearl and Johnson described, on experimental model and then human material, the existence of a complex subcutaneous vascular network located between loose adipose tissue and dense connective tissue that connects the large subcutaneous vessels, the vertical perforators, and the subdermal plexus [36]. Regarding these aspects, they believe that it is possible to determine the optimal flaps for elevation.

The results of our study show that it is not possible using classic research to observe the entire vascular network of the SMAS. Special IHC techniques show us that the vascular network is present in the SMAS even where the muscle fibers are missing—that is, in the retaining ligaments. 

Venous and lymphatic vascularization follows pathways similar to those of the arteries but in the opposite direction. With advancing age, a decrease in blood supply is observed through the rarefaction and fragility of the terminal capillaries, the reduction of blood flow, and the elasticity of the vascular wall. This reduction in vascularization is most often accelerated by arteriosclerosis and smoking [37].

The cervicofacial unit of the cutaneous-musculoaponeurotic, preserved in the case of sub-musculoaponeurotic detachment, is dissociated in the case of subcutaneous detachment [38]. It is the most dissociated in multiplane lifting [39]. The “eutrophic” effect of the classic subcutaneous lifting is an illusion because the subcutaneous lifting is responsible for trophic disturbances in the skin. The zygomatic and mandibular ligaments firmly attach the bones to the dermis. The zygomatic ligament is extremely important in rhytidectomy, and it must be released to allow the mobilization of tissues from the middle part of the face. Unfortunately, the release of this ligament can be dangerous because there are branches of the facial nerve nearby. The following are the auriculoplatysmal ligament and the masseteric cutaneous ligament. The masseteric cutaneous ligament is often topographically associated with multiple branches of the facial nerve.

Ligaments, tendons, and ligamentous adhesions are considered paucivascular/avascular areas. Older studies of the vasculature of the SMAS suggest that it is entirely an avascular layer, but clinical studies show that the incidence of trophic disorders is much higher with subcutaneous dissection. Imaging (CT) research by Schaverien [40] shows that in composite facelifts, the preauricular region has poorer perfusion compared to subcutaneous dissection, although much of this region is excised in rhytidectomy. The lateral facial flap is predominantly perfused from the perforators of the transverse facial artery, and its ligation reduces perfusion in the preauricular region.

Using multiple methods of investigation on fresh cadavers (injection with ink and latex, dissection, and a radiographic examination of the barium-injected sections), 11 vascular territories are described in the face and scalp. The arterial vasculature has three main arrangements. The first one is dense with small and numerous perforating musculocutaneous arteries, which ensure the nutrition of the anterior part of the face (facial infraorbital arteries). The second one is wide with rare fasciocutaneous perforators, which ensure the nutrition of the lateral part of the face (branches from the transverse facial, submental, and zygomatico-orbital arteries). The third arrangement has small, with dense perforating fasciocutaneous arteries that provide nutrition to the scalp (occipital, superficial temporal, and posterior auricular arteries) [34,41].

The attachment ligaments of the superficial fascia to the skeleton compartmentalize the face into several distinct topographic regions and fat compartments. Three of these are at the level of the chin and subzygomatic region: the chin, the side of the chin, and the subzygomatic. In addition to these, we can list the regions of the lower, upper, subtemporal, and frontal eyelids. The ligaments existing between the deep face of the superficial fascia in these regions and the facial skeleton of the mentioned regions “quarantine the movements determined by the contraction of the muscles, at least in young people so that they will not transmit the movements of the superficial fascia to the neighboring regions” [42,43,44].

It is noteworthy that the fibro-adipose adhesions between the SMAS, the deep fascia, and the periosteum limit the mobility and maintain the firmness and gravitational independence of the superficial layers and are the only ones of this type present in the region above. This makes the supporting elements of the SMAS play the most important role in delaying the appearance of the phenomenon known as facial aging. This phenomenon consists in the appearance of wrinkles, and when the supporting elements become loose, the dermis moves in the direction of gravity [13,30,45].

For this reason, the temporal region is the place of choice for performing a facelift that takes advantage of the existence of the SMAS by re-tensioning these adhesions, septa, and ligaments. This is clearly demonstrated by the efficiency of the surgical maneuver, especially after the prior sectioning of the temporal ligaments.

The topographic location of the perforating arteries from the transverse facial, zygomatic orbital, supratrochlear, supraorbital, and superficial temporal artery can be performed and has been shown to be statistically predictable in 95% of cases. Therefore, it is possible to imagine aesthetic or reconstructive surgery techniques based on the knowledge of these arteries.

These statistic results show a relatively constant expression of Collagen III in each of the regions. Meanwhile, the expressions of IHC markers for blood vessels in the endothelium and muscle fibers are highly variable. This means each of the investigated regions are characterized by specific amounts and distributions of blood vessels and muscle fibers.

As a result, we can reject the null hypothesis that all groups have identical mean ranks. At least one group has a mean rank that differs from the others. Additionally, if the distributions of the three anatomical regions share the same shape, we can conclude that the medians differ. The significant Kruskal–Wallis test suggests that at least one sample stochastically dominates another. However, the test does not specify where this dominance occurs or for how many group pairs it applies. Since the Kruskal–Wallis test is a non-parametric method, it does not assume a normal distribution of residuals. Consequently, the null hypothesis is rejected, indicating that the three anatomical region categories do not have the same proportions and the distribution is not uniform.

In an asymmetric distribution skewed to the left (with negative skewness, as indicated by a Skewness statistic of −0.844 for the variables Collagen III and ICAM-2, as shown in Table 5), high scores are more prevalent (denoted by 3+ large amounts/strong intensity). In this scenario, the mode represents the rightmost value in the dataset, and the median exceeds the mean. Clearly, the median serves as the point that divides the ordered data series into two equal parts, and if high scores dominate the distribution, then low scores are considered outliers. It is recognized, through an examination of the accuracy of central tendency measures, that in a dataset featuring extremely low scores, the mean tends to be influenced by them. Consequently, in such a distribution, the relationship is as follows: Mo > Me > m This pattern characterizes a negatively skewed distribution.

In an asymmetric distribution to the right (positive skewness distribution, the Skewness statistic has the value of (0.358) for the MyoH2 variable, according to Table 5), small scores predominate (1+ small amounts/low intensity). In this case, the mode is the leftmost value in the data string, and the median is greater than the mean. Clearly, the median divides the ordered string of data into two equal parts, and if low scores dominate the distribution, then high scores are considered outliers. Through an analysis of the precision of central tendency indicators, we understand that in a dataset where extremely high scores are present, the mean tends to be influenced by them. This relationship is graphically illustrated in Figure 16, and in such a distribution, the relationship is as follows: Mo < Me < m. This is the characteristic relationship of a positively skewed distribution [46,47].

The verification of the normality of the distribution of the results obtained for each variable was conducted using graphical and statistical methods in the SPSS Statistics Version 20 program. Non-parametric tests, specifically the Kolmogorov–Smirnov test and the Shapiro–Wilk test, were employed. These tests led to the conclusion that the experimental data for each variable included in the study do not follow a normal distribution.

Since the significance level (Sig.) of 0.000 is less than 0.05 for both statistical tests, as shown in Table 2, the null hypothesis is rejected.

Modern facelift techniques are oriented towards less extensive variants of a facelift, such as the S-lift, mini facelift, neck lift, or endoscopic facelift. Each case of facial plastic and/or reconstructive surgery must be individualized, and the benefits brought to the patient must be weighed [8].

The results of the micro-CT study show us the presence of a vascular network inside the SMAS but also provide us details about it [48].

Unlike other vascular territories studied, at the level of the retaining ligaments, the vascular network does not follow any fibrous pathways and is not ensheathed. At the level of each of the studied regions, we found blood vessels with a transversal disposition to the skin, whose existence has not been known until now.

Their tortuous path can indicate that they represent a vascular network specific to the SMAS, and this can form anastomosis with the branches that cross the thickness of the SMAS in a plane perpendicular to the skin.

To collect biological samples during facial lifting, involving the SMAS allows the analysis of the biomarkers studied by us in this study. This method can become a follow-up protocol for evaluating the quality and sustainability over time of the performed cosmetic procedure. This is possible via correlating the results of the study of these biomarkers with the determination of adjacent serological markers such as cytokines, myeloperoxidase, and chaperones [49,50,51].

These results may be of particular importance in the reevaluation of techniques to stimulate the healing processes of accidental and postoperative skin wounds, nonsurgical facial rejuvenation, and facial plastic reorientation and reconstructive surgery techniques toward regenerative and functional SMAS surgery.

## 5. Conclusions

The presence of a vascular network in the SMAS, which presents clear regional individualities, obliges us to use molecular techniques to evaluate the morphology, physiology, and topography of these blood vessels. The limitations of this study are the relatively small number of cases studied and the technical limitations of the study protocol.

## Figures and Tables

**Table 1:** Descriptive statistics for independent variables.

Variable	Descriptive Statistics		Statistics	Std. Error
Collagen III	Mean		2.51	0.100
	95% Confidence Interval for Mean	Lower Bound	2.31	
		Upper Bound	2.72	
	5% Trimmed Mean		2.57	
	Median		3.00	
	Variance		0.368	
	Std. Deviation		0.607	
	Minimum		1	
	Maximum		3	
	Range		2	
	Interquartile Range		1	
	Skewness		−0.844	0.388
	Kurtosis		−0.201	0.759
ICAM-2	Mean		2.46	0.107
	95% Confidence Interval for Mean	Lower Bound	2.24	
		Upper Bound	2.68	
	5% Trimmed Mean		2.51	
	Median		3.00	
	Variance		0.422	
	Std. Deviation		0.650	
	Minimum		1	
	Maximum		3	
	Range		2	
	Interquartile Range		1	
	Skewness		−0.844	0.388
	Kurtosis		−0.201	0.759
MyoH2	Mean		1.68	0.103
	95% Confidence Interval for Mean	Lower Bound	1.47	
		Upper Bound	1.88	
	5% Trimmed Mean		1.64	
	Median		2.00	
	Variance		0.392	
	Std. Deviation		0.626	
	Minimum		1	
	Maximum		3	
	Range		2	
	Interquartile Range		1	
	Skewness		0.358	0.388
	Kurtosis		−0.582	0.759
TSMAS (µ)	Mean		40,683.43	2130.788
	95% Confidence Interval for Mean	Lower Bound	36,361.99	
		Upper Bound	45,004.87	
	5% Trimmed Mean		40,387.92	
	Median		31,569.00	
	Variance		167,989,503.5	
	Std. Deviation		12,961.076	
	Minimum		29,578	
	Maximum		57,108	
	Range		27,530	
	Interquartile Range		27,274	
	Skewness		0.512	0.388
	Kurtosis		−1.824	0.759
TAT (µ)	Mean		26,048.65	235.742
	95% Confidence Interval for Mean	Lower Bound	25,570.54	
		Upper Bound	26,526.76	
	5% Trimmed Mean		26,015.51	
	Median		25,068.00	
	Variance		2,056,247.290	
	Std. Deviation		1433.962	
	Minimum		24,791	
	Maximum		27,900	
	Range		3109	
	Interquartile Range		2961	
	Skewness		0.512	0.388
	Kurtosis		−1.822	0.759
BVD (µ)	Mean		2834.00	158.484
	95% Confidence Interval for Mean	Lower Bound	2512.58	
		Upper Bound	3155.42	
	5% Trimmed Mean		2844.86	
	Median		2661.00	
	Variance		929,336.167	
	Std. Deviation		964.021	
	Minimum		1516	
	Maximum		3962	
	Range		2446	
	Interquartile Range		2337	
	Skewness		−0.106	0.388
	Kurtosis		−1.491	0.759
N (µ)	Mean		3.24	0.390
	95% Confidence Interval for Mean	Lower Bound	2.45	
		Upper Bound	4.03	
	5% Trimmed Mean		3.10	
	Median		2.00	
	Variance		5.634	
	Std. Deviation		2.374	
	Minimum		1	
	Maximum		8	
	Range		7	
	Interquartile Range		5	
	Skewness		0.919	0.388
	Kurtosis		−0.734	0.759
L (µ)	Mean		39,493.89	1385.884
	95% Confidence Interval for Mean	Lower Bound	36,683.19	
		Upper Bound	42,304.59	
	5% Trimmed Mean		39,565.91	
	Median		40,289.00	
	Variance		71,064,927.16	
	Std. Deviation		8430.002	
	Minimum		28,979	
	Maximum		48,700	
	Range		19,721	
	Interquartile Range		19,438	
	Skewness		−0.196	0.388
	Kurtosis		−1.670	0.759

**Table 2:** Normality tests for independent variables.

Variables	Kolmogorov–Smirnov			Shapiro–Wilk		
	Statistic	df	Sig.	Statistic	df	Sig.
Collagen III	0.356	37	0.000	0.711	37	0.000
ICAM-2	0.338	37	0.000	0.732	37	0.000
MyoH2	0.292	37	0.000	0.762	37	0.000
TSMAS (µ)	0.380	37	0.000	0.655	37	0.000
TAT (µ)	0.366	37	0.000	0.667	37	0.000
BVD (µ)	0.242	37	0.000	0.811	37	0.000
N (µ)	0.271	37	0.000	0.791	37	0.000
L (µ)	0.236	37	0.000	0.777	37	0.000

**Table 3:** Ranks.

Variable	Anatomical Region	N	Mean Rank
Collagen III	pretragal	10	12.20
	periorbital	14	20.18
	preauricular	13	22.96
	Total	37	
ICAM-2	pretragal	10	15.60
	periorbital	14	19.61
	preauricular	13	20.96
	Total	37	
MyoH2	pretragal	10	16.50
	periorbital	14	18.50
	preauricular	13	21.46
	Total	37	
TSMAS (µ)	pretragal	10	5.50
	periorbital	14	30.50
	preauricular	13	17.00
	Total	37	
TAT (µ)	pretragal	10	5.60
	periorbital	14	30.50
	preauricular	13	16.92
	Total	37	
BVD (µ)	pretragal	10	5.50
	periorbital	14	30.50
	preauricular	13	17.00
	Total	37	
N (µ)	pretragal	10	32.50
	periorbital	14	8.75
	preauricular	13	19.65
	Total	37	
L (µ)	pretragal	10	18.50
	periorbital	14	30.50
	preauricular	13	7.00
	Total	37	

**Table 4:** Statistics ^a,b^.

	Collagen III	ICAM-2	MyoH2	TSMAS (µ)	TAT (µ)	BVD (µ)	N (µ)	L (µ)
Chi-Square	7.668	1.850	1.548	31.801	31.614	31.805	29.802	31.808
df	2	2	2	2	2	2	2	2
Asymp. Sig.	0.022	0.397	0.461	0.000	0.000	0.000	0.000	0.000

^a^. Kruskal–Wallis Test; ^b^. Grouping variable: Anatomical region.

**Table 5:** Descriptive statistics for questionnaire items.

Variable	Mean	Std. Deviation	Skewness (Eroare Standard)	Kurtosis (Eroare Standard)	$\left|Z_{Skewness}\right|$	$\left|Z_{Kurtosis}\right|$
Collagen III	2.51	0.607	−0.844 (0.388)	−0.201 (0.759)	2.18	0.515
ICAM-2	2.46	0.650	−0.844 (0.388)	−0.201 (0.759)	2.08	0.657
MyoH2	1.68	0.626	0.358 (0.388)	−0.582 (0.759)	1.03	0.876
TSMAS(µ)	40,683.4	12,961.08	0.512 (0.388)	−1.824 (0.759)	1.32	2.40
TAT (µ)	26,048.7	1433.962	0.512 (0.388)	−1.822 (0.759)	1.32	2.40
BVD (µ)	2834.0	964.021	−0.106 (0.388)	−1.491 (0.759)	0.27	1.96
N (µ)	3.24	2.374	0.919 (0.388)	−0.734 (0.759)	2.37	0.97
L (µ)	39,493.9	8430.002	0.196 (0.388)	−1.670 (0.759)	0.51	2.20

## Data Availability

Data are contained within the article.
